# Ethacrynic acid is an inhibitor of human factor XIIIa

**DOI:** 10.1186/s40360-022-00575-5

**Published:** 2022-06-01

**Authors:** Srabani Kar, Kayla Vu, Madhusoodanan Mottamal, Rami A. Al-Horani

**Affiliations:** 1 Division of Basic Pharmaceutical Sciences, College of Pharmacy, Xavier University of Louisiana, 1 Drexel Drive, New Orleans, LA 70125-1089 USA; 2 RCMI Cancer Research Center & Department of Chemistry, Xavier University of Louisiana, New Orleans, LA 70125 USA

**Keywords:** Ethacrynic acid, Factor XIIIa, Irreversible inhibitor, Bleeding, Anticoagulant

## Abstract

**Background:**

Ethacrynic acid (EA) is a loop diuretic that is approved orally and parenterally to manage edema-associated diseases. Nevertheless, it was earlier reported that it is also associated with bleeding upon its parenteral administration. In this report, we investigated the effects of EA on human factor XIIIa (FXIIIa) of the coagulation process using a variety of techniques.

**Methods:**

A series of biochemical and computational methods have been used in this study. The potency and efficacy of human FXIIIa inhibition by EA was evaluated using a bisubstrate-based fluorescence trans-glutamination assay under near physiological conditions. To establish the physiological relevance of FXIIIa inhibition by EA, the effect on FXIIIa-mediated polymerization of fibrin(ogen) as well as the formation of fibrin(ogen) – α_2_-antiplasmin complex was evaluated using SDS-PAGE experiments. The selectivity profile of EA against other coagulation proteins was assessed by evaluating EA’s effect on human clotting times in the activated partial thromboplastin time (APTT) and the prothrombin time (PT) assays. We also used molecular modeling studies to put forward a putative binding mode for EA in the active site of FXIIIa. Results involving EA were the average of at least three experiments and the standard error ± 1 was provided. In determining the inhibition parameters, we used non-linear regression analysis.

**Results:**

FXIIIa is a transglutaminase that works at the end of the coagulation process to form an insoluble, rigid, and cross-linked fibrin rich blood clot. In fact, inhibition of FXIIIa-mediated biological processes has been reported to result in a bleeding diathesis. Inhibition of FXIIIa by EA was investigated given the nucleophilic nature of the thiol-containing active site of the enzyme and the Michael acceptor-based electrophilicity of EA. In a bisubstrate-based fluorescence trans-glutamination assay, EA inhibited FXIIIa with a moderate potency (*IC*_*50*_ ~ 105 µM) and efficacy (∆Y ~ 66%). In SDS-PAGE experiments, EA appears to significantly inhibit the FXIIIa-mediated polymerization of fibrin(ogen) as well as the formation of fibrin(ogen) – α_2_-antiplasmin complex which indicates that EA affects the physiological functions of FXIIIa. Interestingly, EA did not affect the clotting times of human plasma in the APTT and the PT assays at the highest concentration tested of 2.5 mM suggesting the lack of effects on the coagulation serine proteases and potentially the functional selectivity of EA with respect to the clotting process. Molecular modeling studies demonstrated that the Michael acceptor of EA forms a covalent bond with catalytic residue of Cys314 in the active site of FXIIIa.

**Conclusions:**

Overall, our studies indicate that EA inhibits the physiological function of human FXIIIa *in vitro* which may potentially contribute to the bleeding complications that were reported with the association of the parenteral administration of EA.

**Supplementary Information:**

The online version contains supplementary material available at 10.1186/s40360-022-00575-5.

## Background

**Fig. 1:**
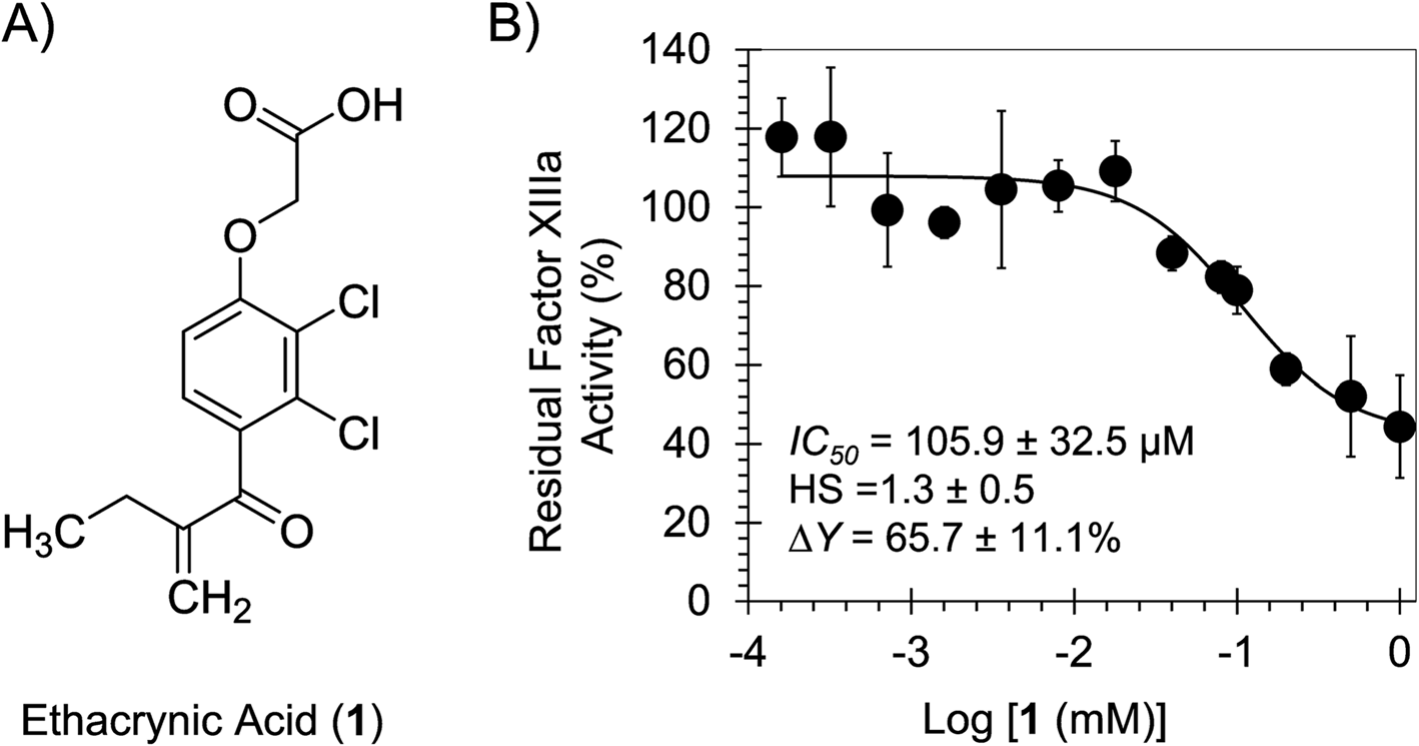
**A** The chemical structure of ethacrynic acid (EA; 1). **B** The inhibition of FXIIIa by EA (●) was measured spectro-fluorometrically through a bisubstrate, fluorescence-based trans-glutamination assay (λ_Ex._ = 360 nm and λ_Em._ = 490 nm) at pH 7.4 and 37 °C. Solid lines represent sigmoidal fits to the data to obtain *IC*_*50*_, HS, Y_M_, and Y_O_ using *Eq. **1*. See details in Methods part. Assay was repeated three times

Ethacrynic acid (EA) is a loop diuretic or high-ceiling diuretic that was approved by FDA in 1967. It is orally used to manage edema associated with hepatic cirrhosis, renal disease, or congestive heart failure. It can also be used for a short-term management of ascites owing to malignancy, idiopathic edema, or lymphedema as well as in a short-term management of hospitalized pediatric patients with congenital heart disease or nephrotic syndrome. It can also intravenously be used when a rapid onset of diuresis is desired as in acute pulmonary edema, or when gastrointestinal absorption is impaired or oral medication is not possible [1, 2]. A finding resulting from comprehensive drug surveillance revealed that a 50 mg-intravenously administered EA (and to some extent orally administered EA) is associated with a clinically significant bleeding, particularly gastrointestinal bleeding [3]. The reported data also suggest that EA may produce bleeding at sites other than the gastrointestinal tract [3]. This observation prompted us to study the *in vitro* effect of EA on human clotting factors, particularly human factor XIIIa (FXIIIa). Chemically, EA (Fig. 1) possesses the electrophilic α,β-unsaturated ketone i.e. Michael acceptor which not only contributes to its diuretic activity via covalently inhibiting Cys-containing proteins in the thick ascending limb of loop of Henle but also to its off target effects.

FXIIIa is a transglutaminase that contributes to several extra- and intra-cellular biological roles. Importantly, the most defined function of human FXIIIa is as a blood coagulation factor. α- and γ-chains of fibrin, factor V, α_2_-antiplasmin (α_2_-AP), thrombin-activable fibrinolysis inhibitor, plasminogen, and plasminogen activator inhibitor-2 have also been reported as substrates for FXIIIa [4–6]. In fact, several studies have established FXIIIa’s importance to hemostasis and has found it to be critical to determine clot properties. Along these lines, it is reported that FXIIIa is essential for maintaining hemostasis by stabilizing the fibrin clot and protecting it from fibrinolytic degradation [7–12]. Therefore, FXIII(a) has been implicated in the risk of atherothrombotic diseases and venous thromboembolism (VTE) [13]. Interestingly, its deficiency results in bleeding diathesis and patients with FXIII deficiency usually need substitution therapy [14, 15]. Biochemically, the catalytic activity of FXIIIa is attributed to its active site that contains the catalytic triad of Cys314, His373 and Asp396. FXIIIa has been found to follow a double displacement mechanism for cross-linking proteins via the formation of an ε-(g-glutamyl) lysine iso-peptide bond. The mechanism has also been reported as a modified ping-pong mechanism that is common among the other transglutaminases [16, 17].

Given the electrophilicity of EA and the nucleophilicity of the active site of FXIIIa, we hypothesized that EA may inhibit FXIIIa affecting the clotting process in a way that potentially, and at least partially, explains the bleeding consequences of using intravenous EA. Using a bisubstrate-based fluorescence trans-glutamination assay, EA was found to inhibit human FXIIIa with a moderate potency (*IC*_*50*_ = 105.9 ± 32.5 µM) and efficacy (∆Y = 65.7 ± 11.1%). In SDS-PAGE experiments, EA appears to dose-dependently inhibit the FXIIIa-mediated polymerization of fibrin(ogen) as well as the formation of fibrin(ogen) – α_2_-AP complex which indicates that EA affects some of the physiological functions of FXIIIa. Interestingly, EA did not affect the clotting times of human plasma in the APTT and PT assays at the highest concentration tested of 2.5 mM suggesting the lack of effects on the coagulation serine proteases and potentially the functional selectivity of EA so as to only affect FXIIIa. Molecular modeling studies demonstrated that the Michael acceptor of EA forms a covalent bond with catalytic residue of Cys314 in the active site of FXIIIa. Overall, these studies indicate that EA inhibits the physiological function of human FXIIIa *in vitro* which may potentially contribute to the bleeding consequences that were reported with the intravenous administration of EA.

## Methods

### Materials

Ethacrynic acid was obtained from Sigma Aldrich (St. Louis, MO). Human plasma thrombin, α_2_-antiplasmin, and FXIIIa were obtained from Haematologic Technologies (Essex Junction, VT). *N,N*-dimethyl-casein, dansyl-cadaverine, and dithiothreitol (DTT) were also from Sigma Aldrich. Dabigatran, rivaroxaban, and AntiF11 (mouse monoclonal antibody from Abnova™) for plasma studies as well as Coomassie Brilliant Blue for gel electrophoresis were from Fisher Scientific (Waltham, MA). Fibrinogen was from Haematologic Technologies. Stock solution of FXIIIa was prepared in 50 mM Tris–HCl, 1 mM CaCl_2_, 100 mM NaCl, 0.1% PEG8000, 0.02% Tween80, and 2 mg/mL *N,N*–dimethylcasein. For the clotting assays, pooled normal human plasma was purchased from George King Bio-Medical (Overland Park, KS). APTT reagent containing ellagic acid, thromboplastin‒D (PT reagent), and 25 mM solution of CaCl_2_ were purchased from Thermo Fisher Scientific. All experiments in this paper were repeated at least two times. For molecular modeling studies, initial structure of FXIIIa (4kty.pdb) was prepared by removing the crystallographic water molecules and adding hydrogen atoms consistent with the physiologic pH of 7 using Maestro 12.4.1. Docking studies were carried out by generating a non-covalent pose using Glide (Schrodinger Suite 2020), and then, by using the covalent docking program (Schrodinger Suite 2020).

### Direct inhibition of human FXIIIa by EA

To evaluate the effect of EA on human FXIIIa, a bisubstrate, fluorescence-based trans-glutamination assay was performed as we reported previously [18–20]. Generally, 1 μL of EA was diluted with 87 μL of pH 7.4 buffer (50 mM Tris–HCl, 1 mM CaCl_2_, 100 mM NaCl, and 2 mg/mL *N,N–*dimethylcasein) and 5 μL dithiothreitol (20 mM) at 37 °C followed by the addition of 2 μL of human FXIIIa (0.3 μM) and incubation for 10 min. The activity of FXIIIa was monitored following the addition of 5 μL of dansylcadaverine (2 mM) by measuring the initial rate of increase in fluorescence emission (λ_Ex._ = 360 nm and λ_Em._ = 490 nm). Relative residual FXIIIa activity at each concentration of the inhibitor was calculated from the ratio of FXIIIa activity in the presence and absence of the inhibitor. Logistic *Eq. **1* was used to fit the concentration dependence of residual FXIa activity so as to obtain the potency (*IC*_*50*_) and efficacy (*ΔY%*) of inhibition.

1 $$Y=Y_0+\frac{Y_M-Y_0}{1+10^{\left(log{\left[I\right]}_0-log{IC}_{50}\right)\left(HS\right)}}$$

In this equation, Y is the ratio of residual FXIa activity in the presence of inhibitor to that in its absence, Y_M_ and Y_0_ are the maximum and minimum possible values of the fractional residual FXIIIa activity, *IC*_*50*_ is the concentration of the inhibitor that leads to 50% inhibition of enzyme activity, and HS is the Hill slope. Y_M_, Y_0_, *IC*_*50*_, and HS values are determined by nonlinear curve fitting of the data.

### Effect of EA on FXIIIa-mediated fibrin(ogen) polymerization

The effect of EA on FXIIIa-mediated fibrin polymerization was further investigated by gel electrophoresis, as reported earlier [18–20]. A solution containing 1.75 mg/ml fibrinogen and 0.9 µg/mL FXIIIa in 50 mM Tris HCl buffer of pH 7.4 containing 10 mM CaCl_2_ was incubated with different concentrations of EA (5 – 5000 µM), and then clotted in the presence of human α-thrombin (1.25 µg/mL). The clots were incubated for 24 h at room temperature before the addition of denaturing buffer of 25 mM NaH_2_PO_4_, 5.7 M urea, 1.9% (w/v) SDS and 1.9% (w/v) DTT, and then incubated overnight at room temperature. Samples were boiled in a water bath for 10 min before centrifugation at 12,000 g at 20 °C for 3 min; the supernatants were examined by SDS-PAGE on homogeneous 10% cross-linked gels and stained with Coomassie Brilliant Blue.

## Effect of EA on FXIIIa-mediated formation of fibrin(ogen) – α_2_-AP complex

This effect was investigated as it was reported previously [21] by western blot assay. A solution containing 1.75 mg/ml fibrinogen and 50 nM FXIIIa in 50 mM HEPES buffer containing 5 mM CaCl_2_ was incubated with different concentrations of EA (100, 500, 1000, 3000, and 5000 µM). After incubation for 10 min, 1.25 µg/mL of thrombin was added to the concoction and further incubated for 30 min at room temperature. At the end of the incubation period, 4 µM of α_2_-AP was added and then the reaction was quenched by using sample reducing buffer containing DDT. The mixture was then fractionated on 10% SDS-PAGE, and then transferred to nitrocellulose membrane, followed by blocking using 5% non-fat dry milk. After vigorous wash with the washing buffer, the membrane was incubated with the primary antibody of human serpinF2/α_2_-AP antibody from R&D systems. The secondary antibody was horseradish peroxidase conjugated anti-goat IgG from R&D systems. The relative positions of bands were confirmed using Western blot analysis.

### Effect of EA on human plasma clotting times

The plasma clotting times; APTT and PT were measured using the BBL Fibrosystem fibrometer (Becton − Dickinson, Sparles, MD), as reported in our previous studies [20, 22]. For the APTT assay, 10 μL of EA (0 – 2500 µM in the clotting cup) was mixed with 90 μL of citrated human plasma and 100 μL of prewarmed APTT reagent (0.2% ellagic acid). After incubation for 4 min at 37 °C, clotting was initiated by adding 100 μL of prewarmed 25 mM CaCl_2_, and the time to clotting was recorded. For the PT assay, thromboplastin-D was prepared according to the manufacturer’s directions by adding 4 mL of distilled water, and then, the resulting mixture was warmed to 37 °C. A 10 μL solution of EA (0 – 2500 µM in the clotting cup) was then mixed with 90 µL of citrated human plasma and was subsequently incubated for 30 s at 37 °C. Following the addition of 200 μL of prewarmed thromboplastin-D preparation, the time to clotting was recorded. In the two assays, about 5 or more concentrations of EA (0 – 2500 µM) were used to establish a concentration vs effect curve. The data were fit to a quadratic trendline, which was used to determine the concentration of EA necessary to double the clotting time. Similar analysis was done for the positive controls (dabigatran [thrombin inhibitor], rivaroxaban [FXa inhibitor], and AntiF11 [FXIa inhibitor]). Clotting times in the absence of EA were also determined in a similar fashion using 10 µL of highly purified water.

### Molecular modeling studies

To identify plausible covalent binding mode of EA on FXIIIa, we carried out covalent docking studies using the covalent docking program developed by Schrodinger (Schrodinger Suite 2020) [23, 24]. Crystal structure of FXIIIa bound to a peptide-like ligand (4kty.pdb) [25] was used for the docking experiment. Initial structure of FXIIIa was prepared by removing the crystallographic water molecules and adding hydrogen atoms consistent with the physiologic pH of 7 using Maestro 12.4.1 Then, the protein molecule was energy minimized with an RMSD cutoff value of 0.3 Å for all heavy atoms. Structure of EA was prepared using the Builder module of Schrodinger followed by energy minimization. The catalytic triad formed by residues Cys314, His373 and Asp396 was used as the ligand binding site for EA. The covalent docking was achieved through Michael addition reaction by properly defining the interacting groups for the ligand and the receptor. Covalent docking feature first docks each ligand to the receptor to generate a non-covalent pose using Glide (Schrodinger Suite) [26]. Then, the covalent bond is formed between the ligand and the receptor (thiol of Cys314) and the resulting ligand pose is refined and scored by performing MM-GBSA calculations with the OPLS/AA force field and GB/SA continuum model. The best-docked structure based on the docking score was selected for further analysis of the binding features of EA to FXIIIa.

## Results

### EA inhibits the trans-glutamination activity of human FXIIIa

Inhibition of human FXIIIa by EA was evaluated by using a modified bisubstrate, fluorescence-based trans-glutamination assay, as described earlier [18–20, 22]. Dansylcadaverine and *N,N*-dimethylcasein were used as two substrates, which upon FXIIIa-mediated conjugation show a marked increase in fluorescence at 490–550 nm (λ_EX_ = 360 nm). To measure the potency and efficacy of EA, the dose-dependence of FXIIIa inhibition was evaluated using the logistic *Eq. **1*. The potency of inhibition refers to the *IC*_*50*_ (x-axis), whereas the efficacy refers to the net change in residual FXIIIa activity (ΔY) (y-axis).

The inhibition profile is shown in Fig. 1B. EA inhibited human FXIIIa with an *IC*_*50*_ of 105.9 ± 32.5 μM and efficacy of 65.7 ± 11.1%. Iodoacetamide (IAA), a nonselective inhibitor of thiol-containing enzymes, was used as a positive control. It inhibited human FXIIIa in previous studies under identical assay condition with an *IC*_*50*_ of 2.9 μM (efficacy =  ~ 100%). Overall, EA is a moderate inhibitor of the trans-glutamination activity of FXIIIa under *in vitro* settings.

### EA inhibits FXIIIa-mediated fibrin(ogen) polymerization

**Fig. 2:**
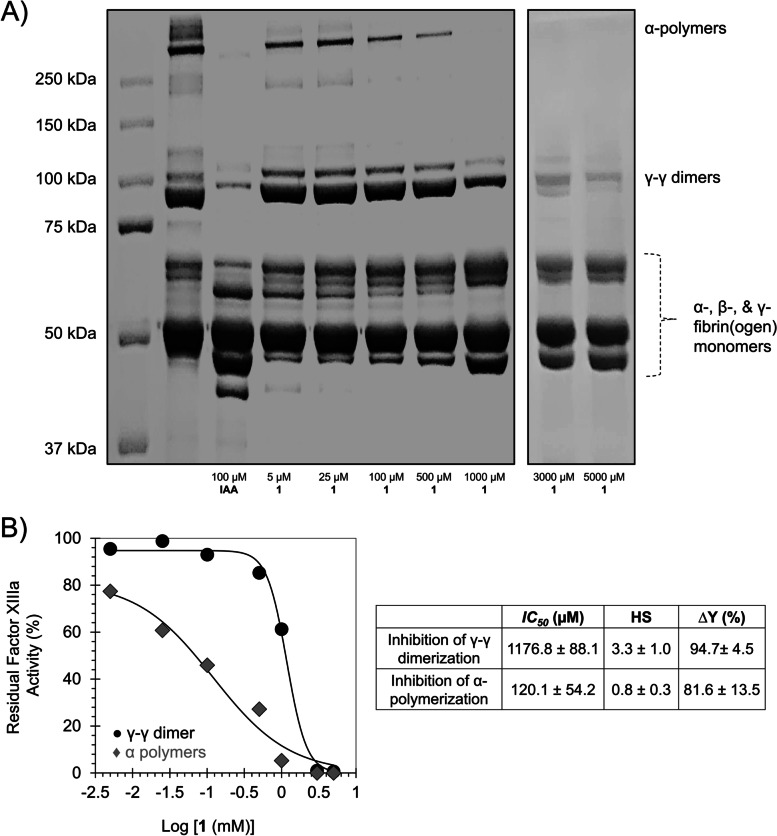
Effect of EA on FXIIIa-mediated fibrin(ogen) polymerization. **A** The effect of EA on FXIIIa-mediated fibrin polymerization was investigated by SDS-PAGE. Different concentrations of EA (5 – 5000 µM) were used. The figure shows that EA concentration dependently inhibited the formation of γ–γ (dimers; about 117 kDa) as well as α–α (larger polymers; > 250 kDa). **B** The dose–response curves for the formation of dimers and polymers from which the inhibition parameters were deduced using *Eq. 1*. EA inhibited the formation of the γ–γ dimers with an *IC*_*50*_ value of ~ 1176.8 µM and an efficacy of ~ 94.7%, yet it was more potent inhibiting the formation of the larger polymers of α – α with and an *IC*_*50*_ value of ~ 120.1 µM and an efficacy of ~ 81.6%. This shows that EA not only inhibiting FXIIIa activity using non-physiological substrates (*N,N*-dimethylcasein and dansylcadaverine) but also using its physiological substrates (α-, β-, and γ- fibrin(ogen)) monomers. Original gels are provided in the supplementary information. The edges were removed to provide better clarity. Experiment was repeated three times

The effect of EA on fibrin(ogen) polymerization was further investigated by SDS-PAGE, as reported earlier for tridegin [18–20]. A solution containing 13 mg/ml fibrinogen and 2.0 µg/mL FXIIIa in Tris-HCl buffer of pH 7.4 containing 10 mM CaCl_2_ was clotted in the presence and absence of human α-thrombin (2.5 µg/mL). The resulting mixture was either incubated with EA (5, 25, 100, 500, 1000, 3000, and 5000 µM) or buffer. The clots were incubated for 24 h at room temperature before the addition of denaturing buffer and then incubated overnight at 25 °C. Samples were boiled in a water bath for 10 min before centrifugation at 12 000 g at 20 °C for 3 min; the supernatants were examined by SDS-PAGE on homogeneous 10% cross-linked gels and stained with Coomassie Brilliant Blue. 100 µM IAA was used as a positive control. The first lane contains the protein markers, whereas the second lane contains the cross-linked fibrin(ogen) formed in the presence of human α-thrombin (Fig. 2A). The lane shows the monomers α-, β-, and γ- bands (~ 50–60 kDa) as well as the cross-linked proteins including the lighter γ-γ dimers (~ 117 kDa) as well as the heavier α-α polymers (> ~ 210 kDa). In one hand, the 100 µM IAA completely inhibited the formation of the cross-linked proteins including the lighter γ-γ dimers as well as the heavier α-α polymers. In the other hand, EA concentration-dependently inhibited the formation of the lighter γ-γ dimers as well as the heavier α-α polymers. Using *Eq. **1*, EA appears to demonstrate different inhibition behavior towards the γ-γ dimerization as well as the heavier α-α polymerization (Fig. 2B). EA inhibited the former with an *IC*_*50*_ of ~ 1177 μM and efficacy of 95%, and it inhibited the latter with an *IC*_*50*_ of ~ 120 μM and efficacy of 82% (Fig. 2B) suggesting that EA is more potent inhibitor of the α-α polymerization of fibrin(ogen). Although such difference in the inhibition behavior of EA toward FXIIIa-mediated dimerization and polymerization of fibrin(ogen) monomers is under investigation, the results indicate that the inhibition activity of EA toward the catalytic activity of human FXIIIa is physiologically relevant.

### EA inhibits FXIIIa-mediated formation of fibrin(ogen) – α_2_-AP complex

**Fig. 3:**
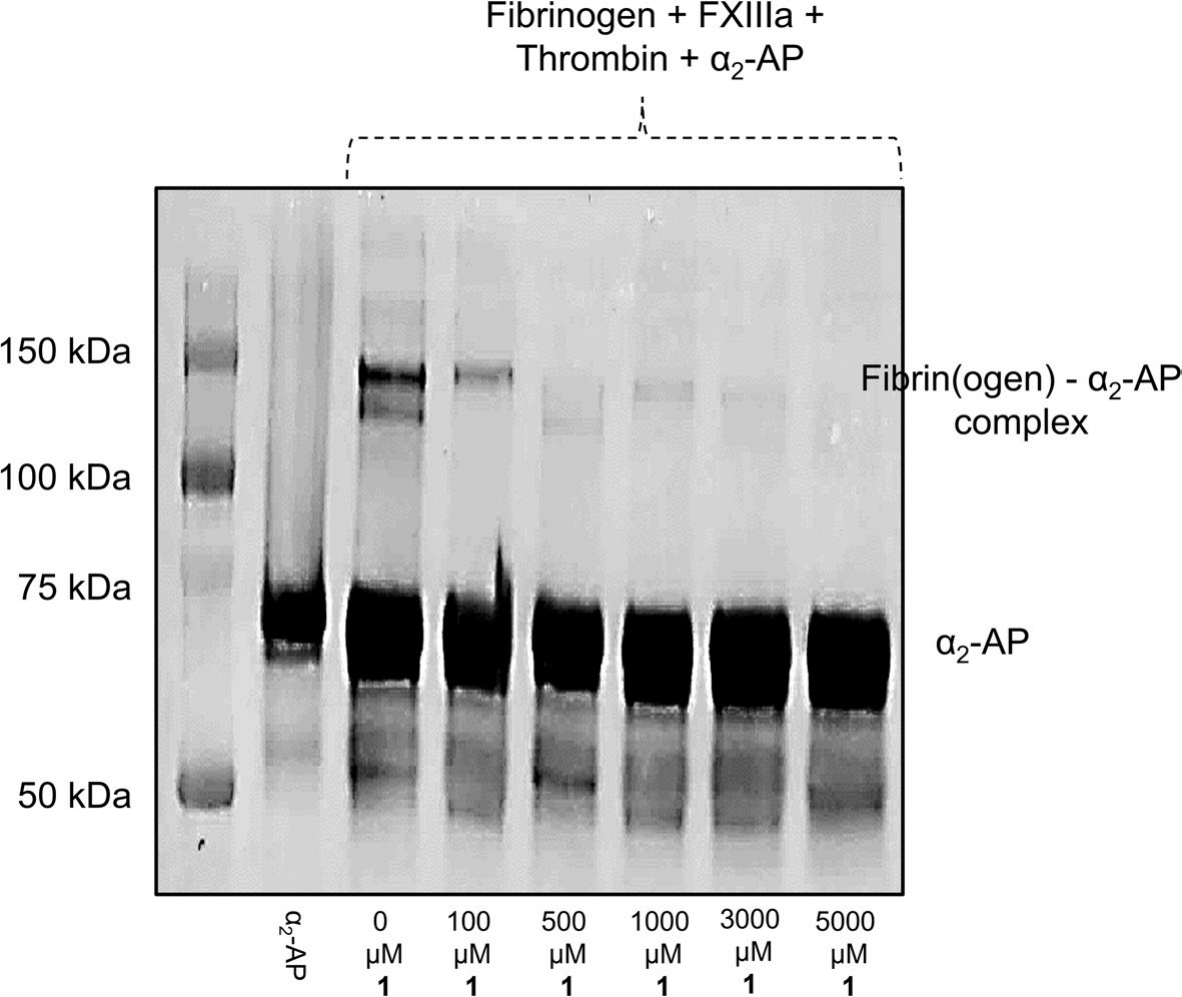
The effect of EA on FXIIIa-mediated formation of fibrin(ogen) – α_2_-AP complex. This effect was investigated by western blot assay in which different concentrations of EA (100, 500, 1000, 3000, and 5000 µM) were used. Evidently, EA inhibited the formation of fibrin(ogen) – α_2_-AP complex at a concentration as low as 100 µM supporting the physiological relevance of the action of EA. In theory, this would mean that the blood clot becomes more susceptible to hydrolysis by the fibrinolytic enzyme plasmin. Original blot is provided in the supplementary information. The edges were removed to provide better clarity. Experiment was repeated three times

An important physiological function of human plasma FXIIIa is to attach the α_2_-AP to fibrin polymers in the blood clot, and thus, it renders the blood clot less susceptible to hydrolysis by plasmin. We investigated the effect of EA on FXIIIa-mediated formation of fibrin(ogen) – α_2_-AP complex by western blot assay, as reported previously [21]. Figure 3 reveals that EA inhibited the formation of fibrin(ogen) – α2-AP complex at a concentration as low as 100 µM supporting the physiological relevance of the action of EA. In theory, this effect makes the blood clot more susceptible to hydrolysis by the fibrinolytic enzyme plasmin, and thus, potentially contributes to the bleeding observed with intravenously administered EA.

### EA does not affect human plasma clotting times

**Fig. 4:**
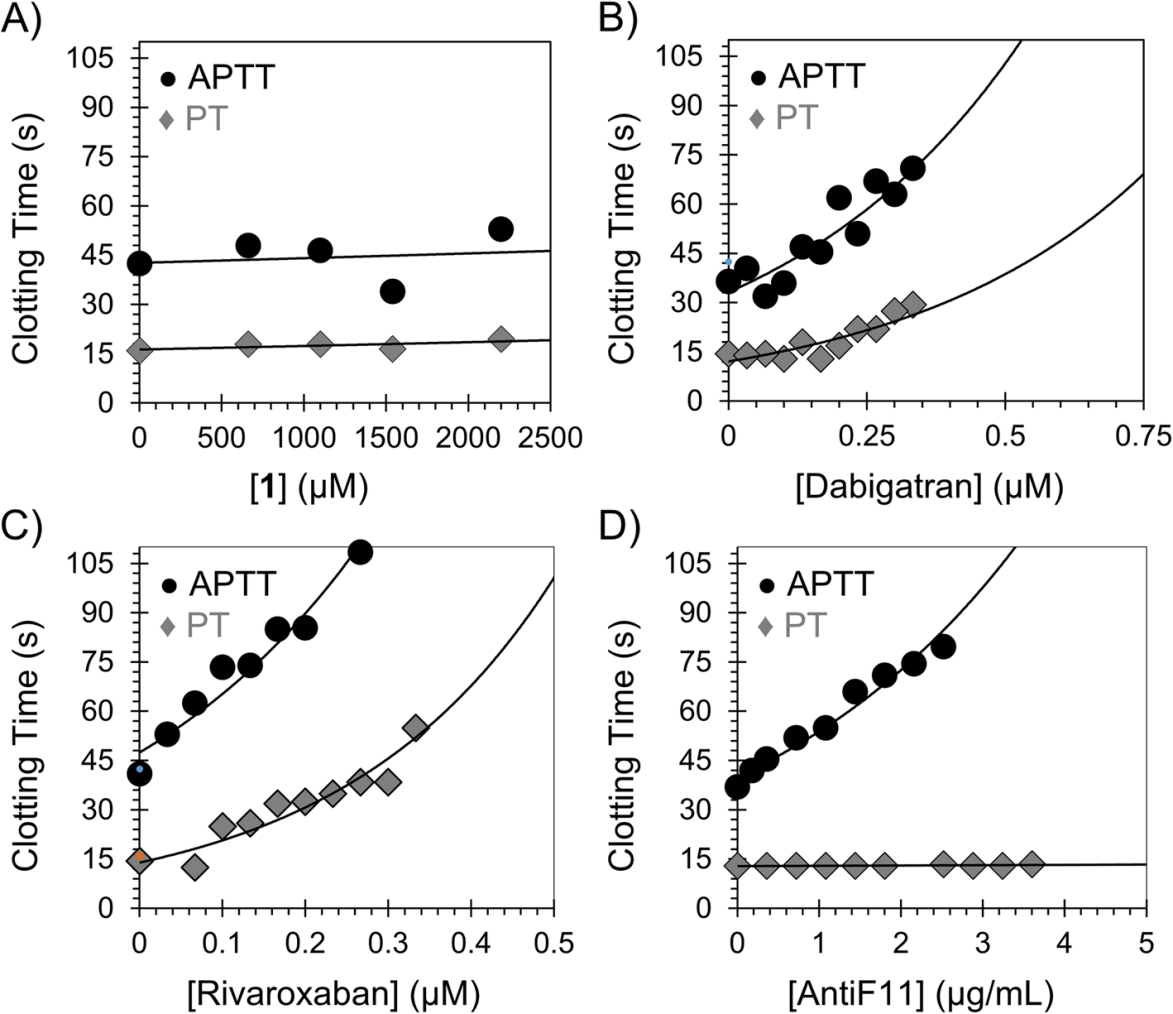
Plasma clotting assays: Activated partial thromboplastin time (APTT) (●) and prothrombin time (♦). **A** The effect of EA (0 – 2500 µM) on APTT and PT. **B** The effect of dabigatran, thrombin inhibitor, (0 – 0.75 µM) on APTT and PT. **C** The effect of rivaroxaban, FXa inhibitor, (0 – 0.5 µM) on APTT and PT. **D** The effect of AntiF11, FXIa inhibitor, (0 – 5 µg/mL) on APTT and PT. Thrombin and FXa inhibitors affect the two times i.e. APTT and PT because they affect the common coagulation pathway, whereas FXIa inhibitor affects only APTT but not PT, which is indicative of an effect on the intrinsic pathway. FXIIIa inhibitors demonstrate similar phenomenon of human FXIIIa deficiency in which APTT and PT are not affected. See details in Methods

To evaluate the effect of EA on other clotting factors most of which are serine proteases, we measured its effect on the plasma clotting times (APTT and PT)  using the BBL Fibrosystem fibrometer (Becton − Dickinson, Sparles, MD), as reported in our previous studies [20, 22]. In one hand, any effect on the APTT (intrinsic coagulation pathway & common coagulation pathway) should reflect an effect on either thrombin, FXa, FIXa, FXIa, or FXIIa. In the other hand, prolongation of the PT (extrinsic coagulation pathway & common coagulation pathway) is indicative of an effect on thrombin, FXa, or FVIIa. In these experiments, we used three positive controls: dabigatran, rivaroxaban, and AntiF11 (Fig. 4A-D). Figures 4A reveals that EA does not affect APTT or PT indicating the lack of an effect on any serine protease enzyme involved in the intrinsic, extrinsic, or common coagulation pathway at the highest concentration tested of 2500 µM. Figure 4B indicates that dabigatran, which is a direct, active site thrombin inhibitor equally affects the APTT and PT. Figure 4C indicates that rivaroxaban, which is a direct, active site FXa inhibitor equally affects the APTT and PT. Figure 4D reveals that the selective AntiF11 antibody only affects FXIa in the intrinsic coagulation pathway (See also Table 1). Important to mention here that EA also lacked any effect on the thrombin time (results are not shown) further supporting its potential selective function at the highest tested concentration. In fact, the behavior of inhibited FXIII(a) (in this case by EA) in term of lacking changes in APTT, PT, and TT is similar to what have been reported for patients with FXIII genetic deficiency [14, 15]. Overall, these results indicate that EA is likely to be a selective inhibitor of the plasma transglutaminase FXIIIa over other plasma serine proteases of the coagulation pathways.

**Table 1:** Effects of EA and other anticoagulants on human plasma clotting times (APTT and PT)

Molecule	Target	APTT_2x_	PT_2x_
EA	FXIIIa	> > 2200 µM	> > 2200 µM
Dabigatran	FIIa	0.32 µM	0.33 µM
Rivaroxaban	FXa	0.19 µM	0.15 µM
AntiF11	FXIa	2.2 µg/mL	> > 3.5 µg/mL

APTT_2x_: The concentration required to double the clotting times of normal human plasma under APTT conditions. PT_2x_: The concentration required to double the clotting time of normal human plasma under PT conditions. Experiment with EA was repeated at least three times, however for the other molecules, experiments were repeated two times because results were highly reproducible with little variability and were consistent with reported values

### EA recognizes Cys314 in the active site of FXIIIa

**Fig. 5:**
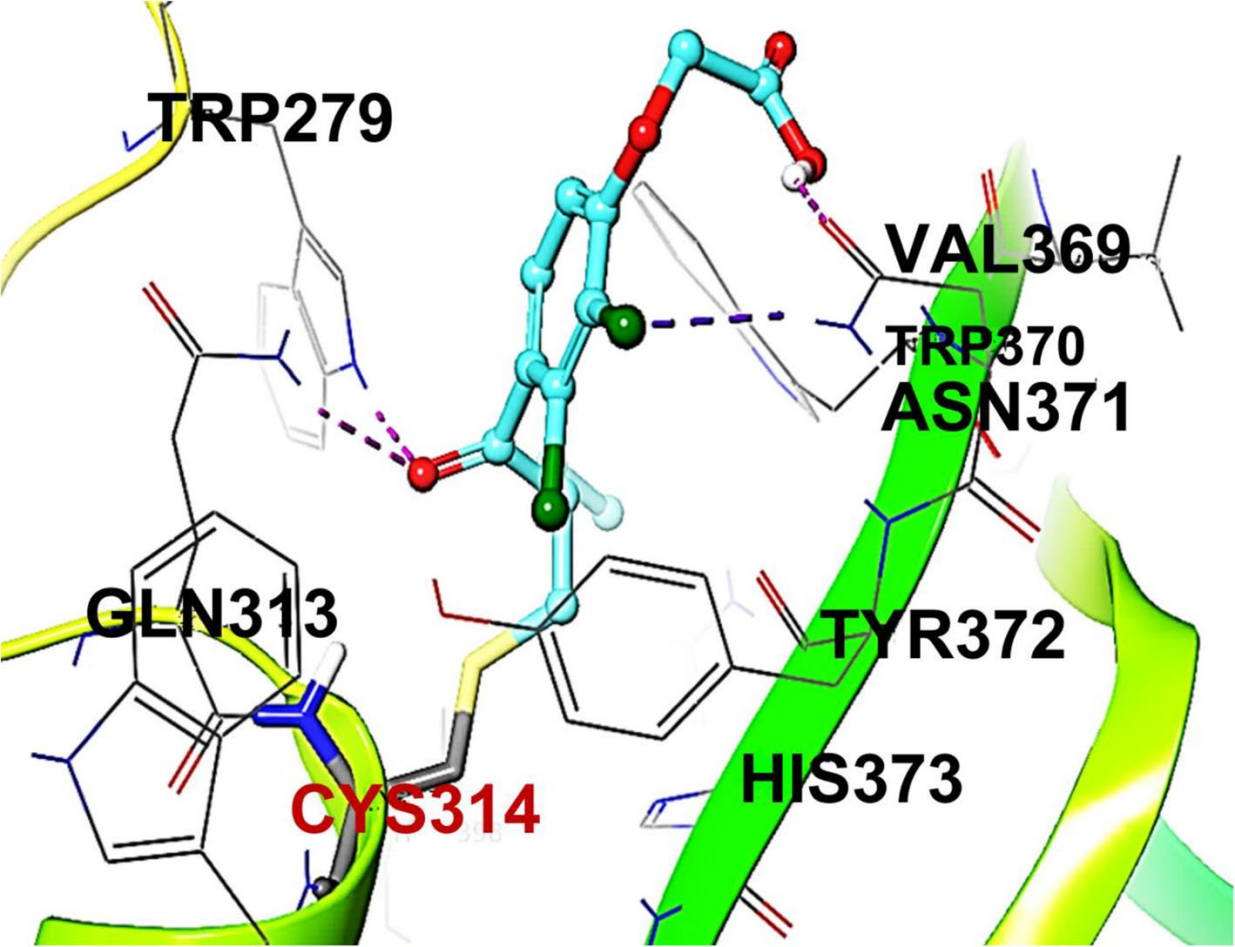
The catalytic domain of FXIIIa is presented as a potential binding site for EA showing the covalent bond between the α,β-unsaturated ketone the catalytic Cys324. Other potential important interactions are H-bonds between the ketone group of EA and the NH-groups of the side chains of Gln313 and Trp279. The carboxylic acid also potentially establishes H-bond with the side chain of the Asn371 residue. The 2-Cl substituent also forms a halogen-H interaction with the side chain of the Asn371 residue. EA is represented as stick and balls. Atoms are represented with the following colors: carbon = cyan, chlorine = green, oxygen = red, nitrogen = blue, and sulfur = yellow. The protein backbone cartoon is represented in green-yellowish color

To identify a plausible binding mode for EA, we performed molecular docking studies by considering the active site of FXIII(a). The rationale for considering this site is that it contains the catalytic triad of which Cys314 appears to be the most critical residue [16, 17]. The covalent docking of EA was achieved through Michael addition reaction by properly defining the interacting groups for the ligand and the enzyme. Covalent docking feature first docks each ligand to the enzyme to generate a non-covalent pose using Glide (Schrodinger Suite) [23, 24]. Then, the covalent bond is formed between the ligand i.e. EA and the enzyme i.e. the thiol of Cys314 of FXIIIa, and the resulting ligand pose is refined and scored by performing MM-GBSA calculations with the OPLS/AA force field and GB/SA continuum model. The best-docked structure based on the docking score was selected for further analysis of the binding features of EA to FXIIIa. Overall. the molecular modeling studies revealed that the catalytic domain of FXIIIa is a potential binding site for EA with a covalent bond forming between the α,β-unsaturated ketone of EA and the catalytic Cys324 of human FXIIIa (Fig. 5). Other potential important interactions are H-bonds between the ketone group of EA and the NH-groups of the side chains of Gln313 and Trp279. The carboxylic acid also potentially establishes H-bond with the side chain of the Asn371 residue. The 2-Cl substituent also forms a halogen-H interaction with the side chain of the Asn371 residue. Although these results are to be experimentally confirmed via crystallography studies and/or mutagenesis studies, however, they further support the concept of FXIIIa inhibition as a potential contributor to the bleeding observed with intravenously administered EA.

## Discussion

A significant association between the administration of EA and the occurrence of gastrointestinal bleeding was observed during a computer monitoring of data obtained in a drug-surveillance program [3]. The collected data also revealed that the drug may produce bleeding at sites other than the gastrointestinal tract [3]. In this study, we have attempted to explain the mechanism of EA-associated bleeding by investigating its potential effect on the coagulation process. We have proposed that EA may act as an irreversible, active site inhibitor of FXIIIa. Inhibiting human plasma FXIIIa is known to produce a fragile blood clot that can be easily digested by fibrinolytic enzymes such as plasmin [7–12]. Not only that but genetic FXIIIa deficiency is also known to lead to bleeding diathesis [14, 15], and thus, it is expected that inhibiting FXIIIa by EA potentially leads to internal bleeding. The rationale for our hypothesis is that the electrophilicity of EA represented by the α,β-unsaturated ketone moiety facilitates an irreversible chemical reaction with the highly nucleophilic Cys314 residue in the catalytic triad of FXIIIa, which is the only transglutaminase among all clotting factors, resulting in its inhibition [16, 17].

Using a bisubstrate-based fluorescence assay, EA was found to inhibit FXIIIa with a moderate potency (*IC*_*50*_ ~ 105.9 µM) and efficacy (∆Y ~ 66%). Furthermore, EA dose-dependently inhibits the FXIIIa-mediated polymerization of fibrin(ogen) as well as the formation of fibrin(ogen) – α_2_-AP complex which indicates that EA affects the physiological functions of FXIIIa. Interestingly, EA did not affect the clotting times of human plasma in the APTT, PT, or TT assays at the highest concentration tested suggesting the lack of effects on the coagulation serine proteases and the selectivity of EA’s action toward human FXIIIa. Molecular modeling studies demonstrated that the Michael acceptor of EA forms a covalent bond with the catalytic residue of Cys314 in the active site of FXIIIa. Considering the pharmacokinetics of intravenously administered EA, it appears that its peak concentrations are of the order of 33 µM [27]. At this concentration, EA partially inhibits FXIIIa (~ 15%). Together, these studies indicate that EA inhibits the physiological function of human FXIIIa *in vitro* which potentially contribute to the bleeding complications known with the intravenous administration of EA.

## Conclusion

We established by various techniques that EA inhibits human FXIIIa *in vitro*. This may partially justify the bleeding complications of intravenously administered EA. As far as the future directions of this discovery, subsequent studies will focus on two dimensions: 1) establishing the above results in a suitable animal model and 2) using EA as a scaffold to develop mechanistically novel anticoagulants that can be used to treat venous thromboembolism (deep vein thrombosis and pulmonary embolism) with substantially lower bleeding tendency compared to the therapeutic doses of currently used anticoagulants including heparins and warfarin [28, 29]. These anticoagulants are either to be used alone or in combination with tissue plasminogen activator [30]. Furthermore, FXIII(a) inhibition has been claimed to provide a promising approach in hypercoagulable patients such as those in the intensive care setting for whom avoiding the formation of (micro)thrombi in the vascular system in sensitive organs is important [30]. This was supported by studies performed in a rabbit sepsis model showing that depletion of FXIII prevents disseminated intravascular coagulation-induced organ damage [31]. Furthermore, FXIIIa inhibitors could also be used clinically to reduce the incidence of acute kidney injury in critically ill patients. Patients that were subjected to continuous renal replacement therapy could also benefit. Such treatment can be better than the current standard of care which remains to be heparins because of the EA’s lower bleeding risk. In fact, EA shows many other advantages as a platform to start with for the development of novel anticoagulants including 1) its ability to immediately promote venous dilatory and relieve pulmonary congestion [2]; 2) the nonpeptide and irreversible nature of its action which guarantees extended duration of action and clinically relevant oral bioavailability [2]; and 3) its 3-step chemical synthetic feasibility [32].

## Supplementary Information

**Additional file 1.** Original gel electrophoresis & Western blots. It includes 4 figures for the original gels and blots.

## Data Availability

They are available from the corresponding author on reasonable request.

## Abbreviations

α_2_-AP: α_2_-Antiplasmin
APTT: Activated partial thromboplastin time
EA: Ethacrynic acid
FXIIIa: Factor XIIIa
PT: Prothrombin time
VTE: Venous thromboembolism
