# Supplementary material for: Ethacrynic acid is an inhibitor of human factor XIIIa
Source: BMC Pharmacol Toxicol. 2022 Jun 1;23:35. doi: 10.1186/s40360-022-00575-5 (PMC9158266; doi:10.1186/s40360-022-00575-5)
Supplement: Supplementary file 1 — Additional file 1. Original gel electrophoresis & Western blots. It includes 4 figures for the original gels and blots. [file 40360_2022_575_MOESM1_ESM.docx]

**Additional file 1:**

**Original gel electrophoresis & Western blots**

**Ethacrynic Acid Is an Inhibitor of Human Factor XIIIa**

Srabani Kar,^1^ Kayla Vu,^1^ Madhusoodanan Mottamal,^2^ and Rami A. Al-Horani^1^*

*^1^Division of Basic Pharmaceutical Sciences, College of Pharmacy, Xavier University of Louisiana,*

*New Orleans LA 70125 USA*

*^2^RCMI Cancer Research Center & Department of Chemistry, Xavier University of Louisiana,*

*New Orleans, LA 70125 USA*

**Figure S1.** Original gel electrophoresis: 1^st^ Experiment of EA (1) effect on FXIIIa-mediated polymerization of fibrin(ogen).

**Figure S2.** Original gel electrophoresis: 2^nd^ Experiment of EA (1) effect on FXIIIa-mediated polymerization of fibrin(ogen).

**Figure S3.** Original gel electrophoresis: 3^rd^ Experiment of EA (1) effect on FXIIIa-mediated polymerization of fibrin(ogen).

**Figure S4.** Original Western blot: Experiment of EA (1) effect on FXIIIa-mediated formation of fibrin(ogen) – α_2_-AP complex.
